# Protein Markers Associated with an ALDH Sub-Population in Colorectal Cancer

**DOI:** 10.4172/jpb.1000412

**Published:** 2016-10-03

**Authors:** Rui Yang, Xinhua Liu, Smathorn Thakolwiboon, Jianhui Zhu, Xiucong Pei, Mingrui An, Zhijing Tan, David M Lubman

**Affiliations:** 1 Department of Surgery, University of Michigan Medical Center, Ann Arbor, Michigan 48109, USA; 2 Experimental Center for Science and Technology, Shanghai University of Traditional Chinese Medicine, Shanghai 201203, China; 3 Department of Medicine, Faculty of Medicine Siriraj Hospital, Mahidol University, Bangkok 10700, Thailand; 4 Department of Toxicology, School of Public Health, Shenyang Medical College, Liaoning 110034, China

**Keywords:** Colon cancer, ALDH, FFPE tissue section, LCM, LC-MS/MS

## Abstract

ALDH has been shown to be a marker that denotes a sub-population of cancer stem cells in colorectal and other cancers. This sub-population of cells shows an increased risk for tumor initiation, metastasis, and resistance to chemotherapy and radiation resulting in recurrence and death. It is thus essential to identify the important signaling pathways related to ALDH1+ CSCs in colon cancer. The essential issue becomes to isolate pure sub-populations of cells from heterogeneous tissues for further analysis. To achieve this goal, tissues from colorectal cancer Stage III patients were immuno-stained with ALDH1 antibody. Target ALDH1+ and ALDH1− cells from the same tissue were micro-dissected using Laser Capture Microdissection (LCM). Captured cells were lysed and analyzed using LC-MS/MS where around 20,000 cells were available for analysis. This analysis resulted in 134 proteins which were differentially expressed between ALDH1+ and ALDH1− cells in three patient sample pairs. Based on these differentially expressed proteins an IPA pathway analysis was performed that showed two key pathways in cell to cell signaling and organismal injury and abnormalities. The IPA analysis revealed β-catenin, NFκB (p65) and TGFβ1 as important cancer-related proteins in these pathways. A TMA validation using immunofluorescence staining of tissue micro-arrays including 170 cases was used to verify that these key proteins were highly overexpressed in ALDH1+ cells in colon cancer tissues compared to ALDH1− cells.

## Introduction

Colorectal cancer (CRC) is the second leading cause of cancer death in the United States [1–3]. Although almost 70% of patients can be operated on with intent to cure, up to 30% of all these patients will relapse within 2–3 years [4]. Survival rates for colon cancer have steadily improved mainly due to a combination of earlier diagnosis and improvements in treatment [5–6]. Nevertheless, an improved understanding of the protein signaling pathways could provide new biomarkers for potential targets of therapeutic and surgical intervention [7–8].

Tissue-based proteome analysis is essential for understanding the signaling pathways in cancer especially when the tissue is from the primary tumor site [9–11]. In particular, formalin-fixed paraffin-embedded (FFPE) samples are routinely used for processing and storage of pathology specimens. However, the analysis of FFPE tissue sections is limited by tissue heterogeneity, where the phenotype pathways need to be associated with specific populations of cells. Indeed there may be several sub-populations of cells that are responsible for different aspects of cancer progression which need to be identified. It thus becomes essential to isolate targeted populations of cells including bulk cancer cells and cancer stem cell populations defined by different surface markers.

Aldehyde dehydrogenase (ALDH) has been identified as a potential universal marker for stem and progenitor cells in epithelial cancers [12]. Recent work has shown that high ALDH activity is associated with self-renewal in several normal and tumor tissues [13–19]. Among human ALDH isoforms, ALDH1 has been demonstrated as a promising new marker for colon cancer stem cells. Recent work has shown that there is reduced expression of ALDH1 in epithelial cells at the base of the normal crypt compared to cancerous colon tissue [12]. In cancer ALDH1-expressing cells are no longer limited to the base of the crypt and can be found throughout the epithelium. ALDH1 plays a major role in the biosynthesis of retinoic acid from retinol, which may modulate stem cell proliferation [20]. Therefore, we used the ALDH1 antibody for immuno-histochemical staining of ALDH+ cells.

In the current study we have undertaken a targeted proteomics study of ALDH1 positive colon cancer tissues. An improved understanding of differentially expressed proteins identified from a sub-population extracted from tissues may provide a means for discovery of potential markers and therapeutic targets as well as enhanced understanding of processes involved in colon cancer. An immuno-histochemical antibody-staining method has been used to detect targeted sub-populations while laser capture microdissection (LCM) has been used for isolating cancer stem cells (CSCs) and bulk cancer cells from a complex tissue [21–23]. These cells were then analyzed by high-resolution mass spectrometry. We detected 134 differentially expressed proteins which were then used with the IPA database to identify important cancer-related proteins that are found in key signaling networks. Three proteins of interest, β-catenin, NFκB and TGFβ1 were identified as being cancer-related proteins in the cell to cell signaling and organismal injury and abnormalities pathways. β-catenin and TGFβ1 were detected by mass spectrometry whereas NFκB was not directly detected; however these proteins were further validated by tissue microarray analysis in a large number of samples demonstrating that they are highly overexpressed in cancer tissues.

## Materials and Methods

### Immunohistochemistry of FFPE human tissues with ALDH1

We used tissue specimens (5 μm thickness) from three patients with colonic adenocarcinoma at stage III (BioChain Institute, Inc. (Newark, CA)). Tumor tissue related data are listed in Supplemental Table S1, Supporting Information. The FFPE slides were processed as described previously [24,25]. Ultimately, the slides were blocked with 2% BSA for 1 h at room temperature and incubated with a mouse anti-human ALDH1A1 monoclonal antibody (1:75 dilution, BD Transduction Laboratories, NJ) overnight at 4°C and washed with PBS three times (10 min each). Then a goat anti-mouse HRP-conjugated secondary antibody (1:200, Vector laboratories, Burlingame, CA) was used to incubate the slides for an hour. The slides were washed again with PBS three times (10 min each), and the immunoreactions (brown) were detected using the DAB Substrate kit (Vector laboratories, Burlingame, CA). Hematoxylin counterstaining was performed for nucleus visualization. The slides were then dehydrated in 70%, 95%, and 100% ethanol for 30 s each, rinsed in xylene for a few seconds, airdried in a fume hood, and then placed in a desiccator to dry completely.

### Immuno-Laser Capture Microdissection (iLCM)

LCM was performed on a Veritas Arcturus LCM System (Arcturus, Molecular Devices, CA), which uses a UV/IR laser arrangement as described in previous work [23]. ALDH1+ and ALDH1− areas were selected and micro-dissected from CRC (n=3) respectively, under a 20× objective. LCM was carried out on 5 tissue sections per patient and 17.0 mm^2^ ALDH1+ surface area was collected with an approximately equal area of ALDH1− as a control. The capture laser parameters were: Power=50–60 mV, Pulse=1.5–2 ms, Spot size=20–30 μm, Intensity=200 mV, and the cutting laser was set as: Spot size=2.0 μm, Laser power=7.0 (low).

### Protein extraction/Digestion by FASP

Proteins from the micro-dissected cells were extracted and digested using the filter-aided sample preparation (FASP) protocol [23,26]. A commercial FASP Protein Digestion Kit (Expedeon Inc., San Diego, CA) was used in this work. The FASP procedure was performed as described previously [23,27]. Enzymatic digestion was performed by adding trypsin (Promega, Madison, MI) in 75 μL of 50 mM NH_4_HCO_3_ to the filter. The protein to enzyme ratio was 100:1. Samples were incubated overnight at 37°C. Released peptides were collected by centrifugation and desalted with ZipTip C_18_ tips (Millipore, Billerica, MA).

### LC-MS/MS analysis

The peptide mixtures were analyzed by LC-MS/MS using an Orbitrap Elite mass spectrometer (Thermo). Chromatographic separation of peptides was as performed previously [23]. The MS instrument was operated in positive ion mode. Survey MS scans (from *m/z* 400–1,800) were acquired in the Orbitrap analyzer with resolution R=120,000 at *m/z* 400, and the top 20 most intense ions were selected for tandem MS analysis by CID in the linear ion trap. The normalized collision energy was set at 35% for MS/MS. Dynamic exclusion was defined enabled: exclusion duration was 60 s, and repeat count was 2.

### Data analysis

All MS/MS spectra were searched against the UniProt human protein database Release 2014_01 by using SEQUEST algorithm incorporated in Proteome Discoverer software version 1.4. The initial maximal mass tolerance in mass mode was set to 10 ppm, whereas fragment mass tolerance was set to 0.8 Da. The maximum false peptide discovery rate was specified as 0.01. Carbamidomethyl of cysteine (C) was set as a fixed modification and oxidation of methionine (M) as a variable modification. Label free quantification was performed using a normalized spectral counting method due to the small number of cells available for analysis. The procedures for this spectral count are as described previously [23].

### Ingenuity Pathway Analysis (IPA)

The molecular function and biological networks of the differentially expressed proteins were analyzed using the IPA software (Ingenuity System, Mountain View, CA). Differentially expressed proteins identified between ALDH1+ and ALDH1− cells in three patients were uploaded into the pathway analysis tool. The uploaded Excel file contains the relevant proteins with their fold change, *p*-value and corresponding primary accession number. The significance value for canonical pathways was calculated using the right-tailed Fisher’s exact test by comparing the number of proteins that were involved in a given function or pathway relative to the total number of occurrences of these proteins in all functional/pathway annotations stored in the Ingenuity Pathway Knowledge Base (IPKB).

### Tissue microarray and double immunofluorescence analysis

The tissue microarrays (TMAs) of FFPE colon cancer tissues and normal tissues were purchased from US Biomax Inc. (Rockville, MD). The TMAs contained 155 cases of cancer tissues of different stages and 15 normal tissues, and the clinical pathologic characteristics are listed in Table 1. The FFPE tissue arrays were dewaxed as described previously [23,24]. The TMAs were treated with citrate buffer at pH6.0 (Invitrogen, Grand Island, NY) and then were blocked from non-specific binding by 2% BSA. To achieve double immunofluorescence staining of ALDH1 and other candidate markers, the mouse anti-ALDH1A1 antibody (BD, 1:75) was mixed with the rabbit anti-β-catenin (Abcam, 1:200) and rabbit anti-TGFβ1 (LifeSpan Biosciences, 1:100), respectively, and the mouse anti-NFκB(p65) antibody (Cell signaling, 1:400) was mixed with the rabbit anti-ALDH1A1 antibody (Abcam, 1:200). The antibody mixture was then incubated with the TMAs overnight at 4°C. Then DyLight 549 anti-mouse IgG (red) and DyLight 488 anti-rabbit IgG (green) (Vector laboratories, Burlingame, CA) were diluted (1:200) and incubated with the TMAs for 1 hr at room temperature. Nuclei visualization was performed by DAPI counterstaining (blue) (1:8,000). The TMAs were finally dehydrated in alcohol and cover-slipped.

### Evaluation of immunofluorescence staining

The overlap of candidate proteins with ALDH1 was investigated by double immunofluorescence on tissue microarrays, which contains 155 colon cancer tissues with clinical stages and 15 normal tissues. IF score was based on the product of the percentage ALDH1+ cells multiplied by stain intensity (0=No staining, 1=Weak, 2=Moderate, 3=Strong) and percentage of stained cells (0=No staining, 1 ≤ 10%, 2=10–50%, 3=51%–80, 4 ≥ 80%) for each specimen. To calculate the overlap, 3 random areas were chosen under 200× magnification. The numbers of ALDH1+ gland cells and the gland cells with positive staining for these markers have been counted. Then we performed statistical analysis of these 155 cases using GraphPad Prism software (Version 6). *p*-value <0.05 was considered as a significant difference between the samples in different stages.

## Results and Discussion

ALDH1 proteins are primarily localized in the various tissues. It has been demonstrated that ALDH1 expression is associated with drug resistance during cancer treatment. ALDH1A1 in particular which is an isotype of ALDH1 has been the key ALDH marker of CSCs including for colon cancer stem and progenitor cells [28]. ALDH1A1 was therefore used as a marker to isolate cancerous human colon stem cells where ALDH1+ and ALDH1− cells were isolated from colon cancer tissue, followed by proteomic analysis. We used MS data to determine pathways to look for significant cancer-related proteins for further validation even if not detected directly by mass analysis.

### Proteomics workflow

**Figure 1:**
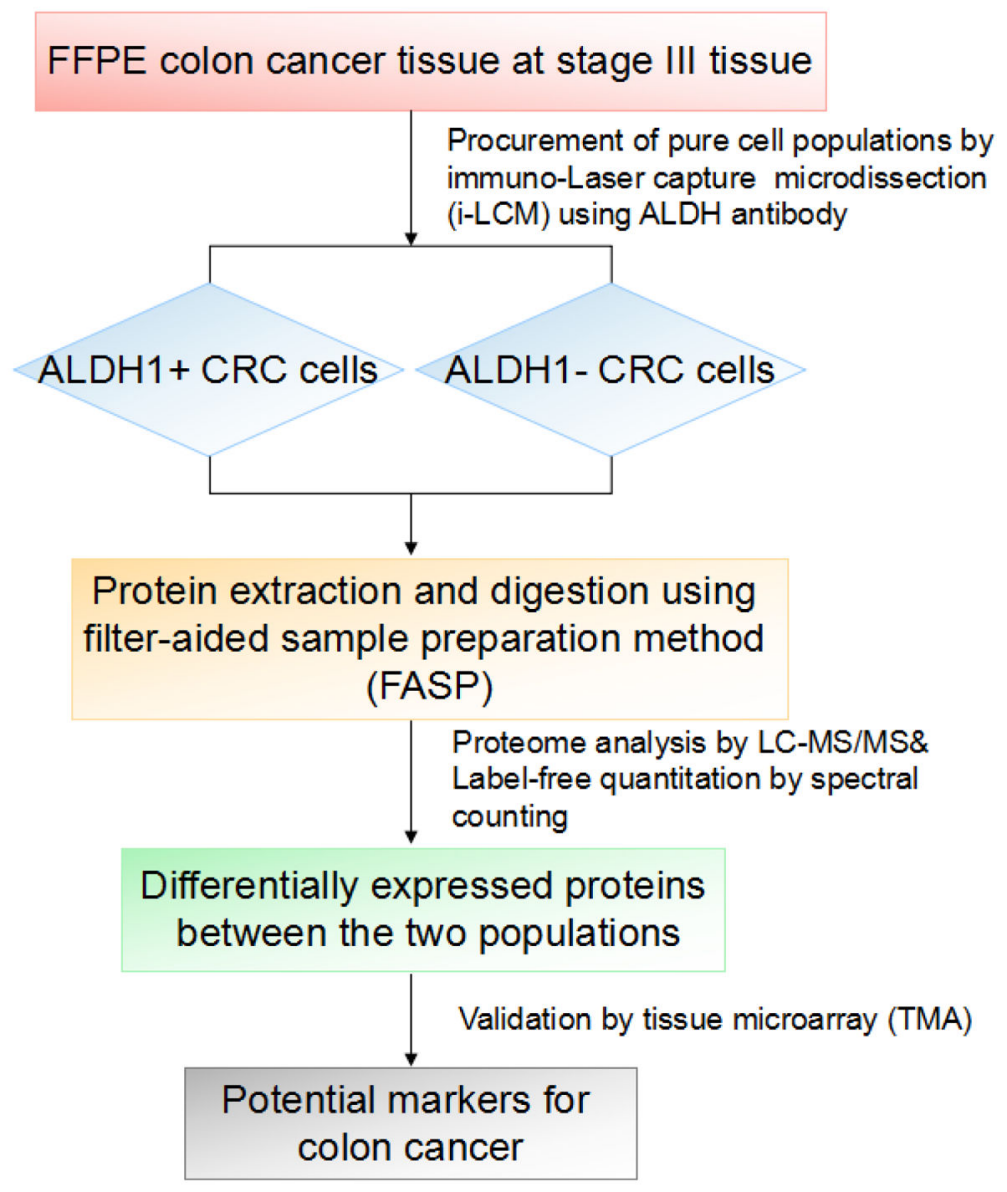
The proteomic workflow used in this study is summarized. We have developed a method of analyzing pure cell subpopulations isolated from FFPE tissue sections combining immunohistochemistry, laser microdissection, and shotgun proteome analysis.

Figure 1 shows the workflow of this study, which is based on analysis of FFPE using immunohistochemical antibody-staining combined with iLCM to target cell populations which can be isolated using laser-capture microdissection (LCM). Single cells were isolated from tissue slices of FFPE blocks. These sections of colon cancer tissue are from three patients. The method used for analysis is a combination of immunohistochemistry, laser microdissection, and shotgun proteome analysis.

Protein analysis is performed using LC-MS/MS-based shotgun proteomics where large numbers of proteins can be identified and quantified for further analysis based on bioinformatics methods and can be validated on tissue microarrays.

### Microdissection of ALDH1+ and ALDH1− cells from FFPE tissues

After ALDH1 immunohistochemistry staining, approximately a 17.0 mm^2^ area was micro-dissected per specimen. We estimate that approximately 20,000 cells were dissected from each specimen; however this is only an estimate since the sizes of cells in tissues are heterogeneous.

### Analysis of low sample amounts

In proteomic MS-based analysis identification and reliable quantification depend on the quality of protein solubilization and digestion. We used the FASP method to analyze low levels of protein. The FASP procedure yields a linear relationship between the volume of micro-dissected tissue and peptides. Using human samples, it was found that 175 nL of micro-dissected tissue yielded about 5–7 μg of peptide material [29]. In this study, 85 nL of tissue (approximately 2 μg peptides) was harvested from each specimen and the tissue was extracted and digested by the FASP method followed by tandem MS analysis. All LC-MS/MS spectra were searched using the SEQUEST algorithm incorporated in Proteome Discoverer software version 1.4 (Thermo) against the UniProt human protein database Release 2014_01. A total of 1927 proteins were identified across the three specimens (Supplemental Table S2).

### Determination of differentially expressed proteins

Significant proteins between ALDH1+ and ALDH1− specimens were identified by label-free shotgun proteomics. To achieve quantification, we normalized the spectral counts identified in each LC-MS/MS run to reduce the variance between samples and replicates. The fold-change was calculated as the ratio of the average normalized spectral count for the target protein between ALDH1+ and ALDH1− cells. The Student’s *t*-test was applied across the six biological specimens (3 technical replicates for each specimen) to calculate the *p*-value to determine the significance of the different protein expressions. The cutoff for differential expression was set at a 2-fold change between ALDH1+ and ALDH1− cells with *p*-value <0.01.

There are 134 proteins identified as commonly differentially expressed between ALDH1+ and ALDH1− in the three patient-matched sample pairs (Supplemental Table S3). Among these common proteins, were 101 proteins overexpressed and 33 under-expressed in ALDH1+ cells compared to ALDH1− cells. It should be noted that ALDH and its isoforms were detected directly by mass spectrometry and are listed in Table S4.

### Signaling pathways

**Figure 2:**
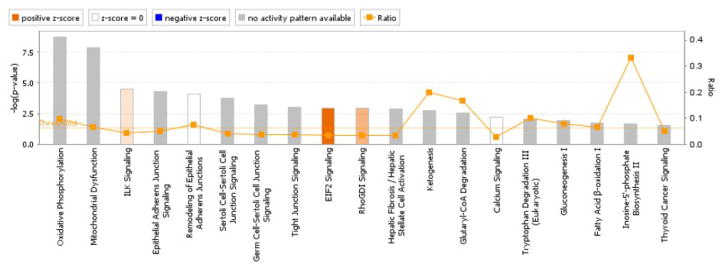
Canonical signaling pathways enriched with commonly differentially expressed proteins between ALDH1+ and ALDH1− cells and ranked by significance in the IPA. A *p*-value threshold of 0.01 is applied.

We input an Excel file containing 134 differentially expressed proteins identified between ALDH1+ and ALDH1− cells in three patient-matched sample pairs into the pathway analysis tool IPA (Ingenuity Systems) to determine the signaling pathways related to the proteome data. IPA produced the most important signaling pathways ranked by significance. Figure 2 shows the significant signaling pathways with *p*-value <0.01. The length of the bars indicates the significance of the signaling pathways of which the differentially expressed proteins are involved.

These enriched pathways shown in Figure 2 can be grouped into three major categories: RNA Post-Transcriptional Modification, Cellular Growth and Proliferation, and Cell-To-Cell Signaling and Interaction. The most prominent signaling pathway, EIF2 Signaling, has a variety of stimuli that modulate eIF2 activities which in turn regulate mRNA translation and effect the initiation of protein synthesis. Gluconeogenesis I is a drug pathway. Glutaryl-CoA Degradation is a common intermediate in the degradation of many different compounds, such as benzoate and benzoyl-CoA, L-lysine, L-tryptophan among others. Fatty Acid β-oxidation I is a very important pathway, especially in colon adenocarcinoma, where many researchers have shown that fatty acids can regulate the colon immune system. Two carbon atoms are removed during every cycle until only two or three remain. If there are even-numbered fatty acids broken down, a two-carbon compound remains as acetyl-CoA. When odd numbers of fatty acids are broken down, a three-carbon residue results in propionylCoA. This is further catabolized by the reactions of proprionate catabolism. Tryptophan Degradation III is involved in endocrine system development and energy production.

### Connectivity network analysis

**Figure 3:**
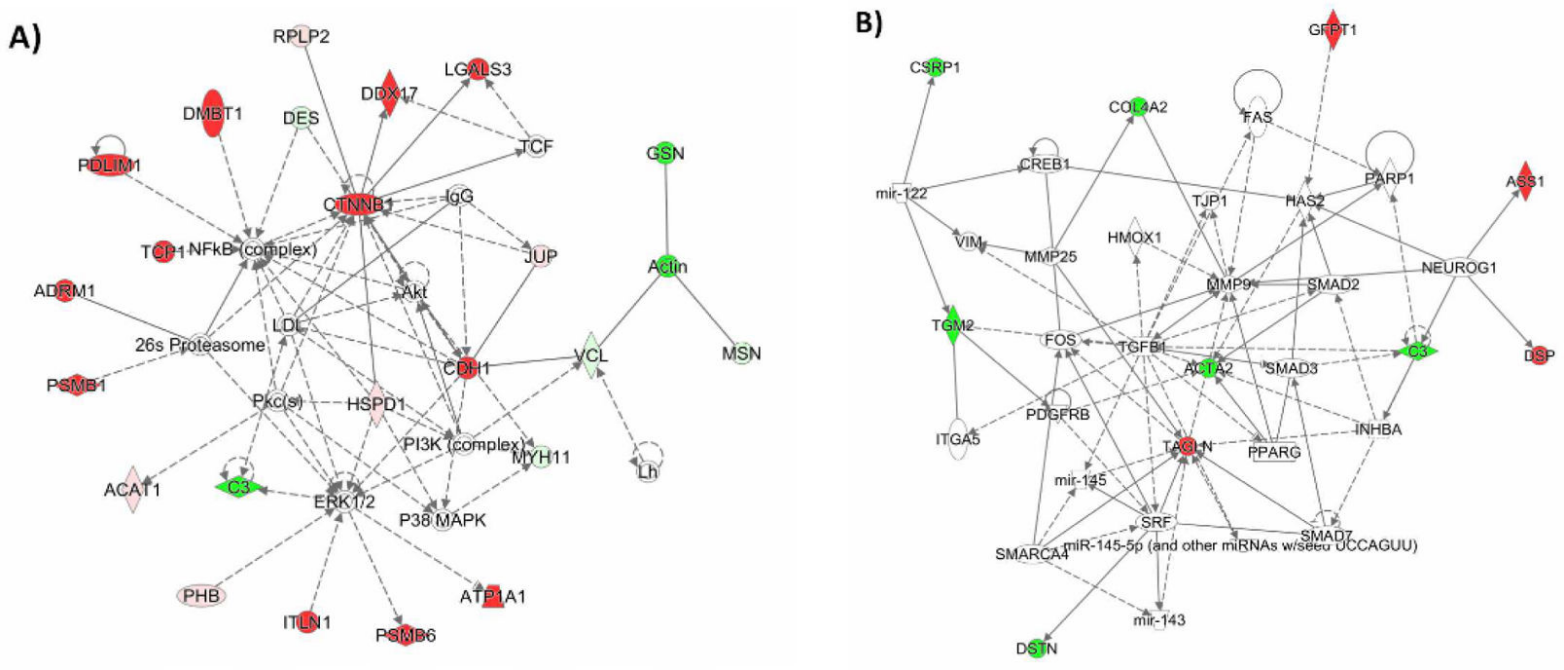
Top two connectivity networks constructed by IPA: (A) Cell-to-Cell Signaling; (B) Organismal Injury and Abnormalities. These networks only consist of differentially expressed proteins derived from the experimental data. Red and green represent over- and under-expression in ALDH1+ cells compared with ALDH1- cells, respectively. White indicates proteins that were not identified as differentially expressed in this study or were not detected by the mass spec analysis but are relevant to the networks.

In order to understand the functional relevance of the proteins which take part in cell regulation, all the potential networks were shown by IPA. Figure 3 shows the two most important networks which are relevant to Cell to Cell Signaling (A), and Organismal Injury and Abnormalities (B). These networks only contain differentially expressed proteins derived from the experimental data. Red represents over-expression and green represents under-expression in ALDH1+ cells compared with ALDH1− cells, respectively. White indicates proteins that were not identified as differentially expressed in this study or were not detected by the mass spec analysis but are relevant to the networks. 40 proteins involved in these networks that we detected by mass spectrometry analysis directly are listed in Supplemental Tables S5 and S6. The fold-change was calculated as the ratio of the normalized spectral abundance of each protein between ALDH1+ and ALDH1− cells in the three patients respectively.

### Immunofluorescence validation of selected potential markers

We used tissue micro-arrays to validate key proteins in our network over a large number of samples since only three patient samples were used in our MS analysis. All the candidates were chosen from the two Networks. β-catenin is in our network A, where this protein is a key factor in a variety of human tumors and is directly detected by mass spectrometry (Table S2). The upregulation of β-catenin always results from the downregulation of E-cadherin [30]. β-catenin expression reflects the aggressiveness of the primary tumor and the depth of invasion [31–33]. β-catenin/Tcf-4 signaling is important in initiating tumorigenesis which has been revealed by the upregulation of β-catenin in the early stage of colon cancer. Additionally, the up-regulation of β-catenin is known to contribute to tumor formation as shown by immunostaining work [34].

**Figure 4:**
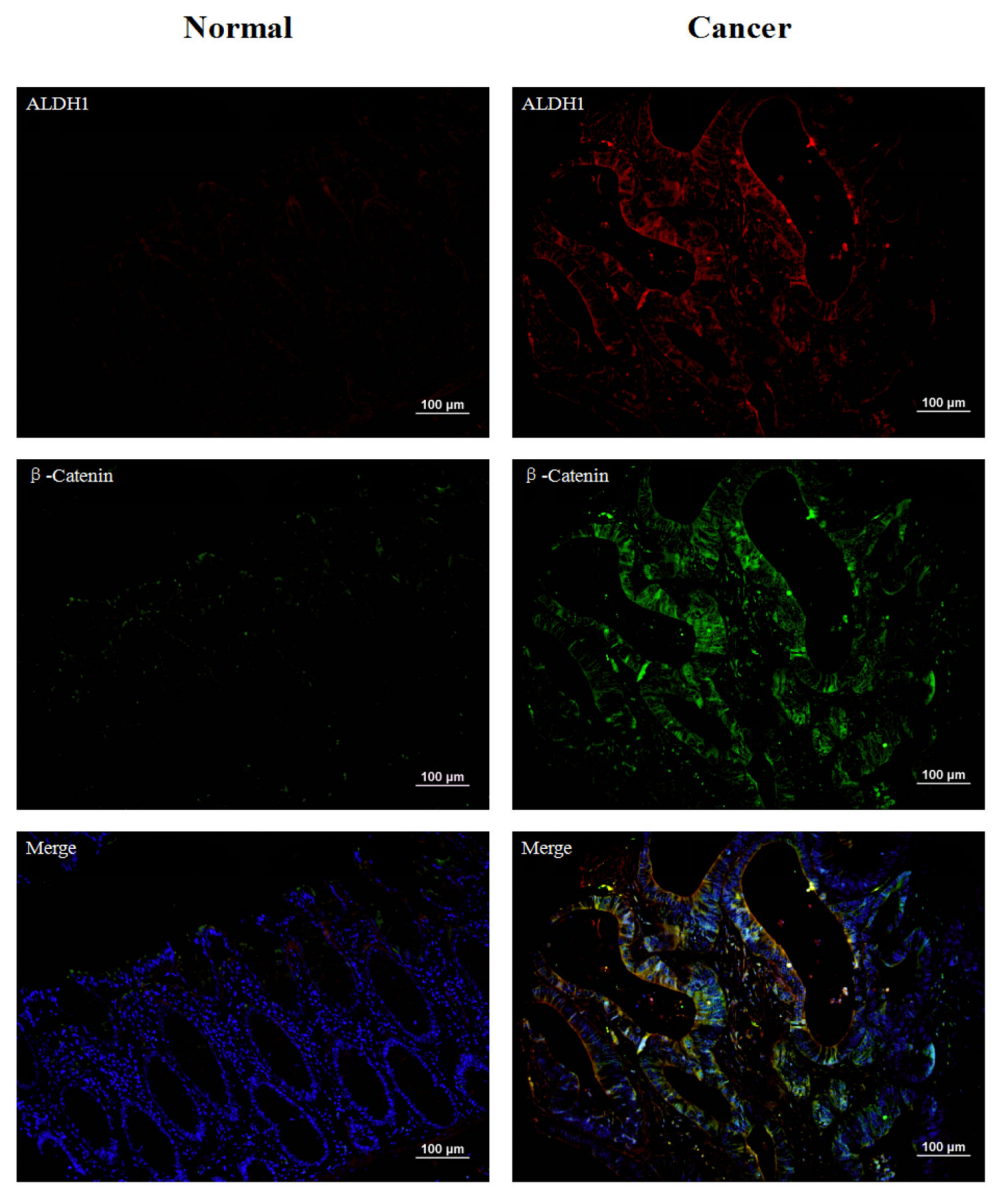
Immunofluorescence double staining with ALDH1 (red) and β-catenin (green) in human normal colon tissue and colon cancer. DAPI represent the nucleus of the cells. Overall, the expressions of ALDH1 and β-catenin are negative in normal tissues. ALDH1 is positive on cell membranes. However, both of them are overexpressed in cancer tissues. β-catenin shows strong nucleus positive staining and is highly overlapped with ALDH1. Magnification 200x, scale bars=100 μm.

We have stained β-catenin in TMAs including 155 cases of different stages and grades by immunofluorescence. The percentage of positive staining reaches 72.9% (Table 2), where the staining images are shown in Figure 4. We detected up to 106 overlapped cases among the 155 cases where there is overlap between ALDH1 and β-catenin staining (Table 3). Three random areas of interest were chosen to count overlapped and total cell numbers from each of 106 overlapped cases. We used the overlapped cell numbers divided by total cell numbers to calculate the percentage of overlapped cells which was found to be 74.8% (Table S6). As in a previous study, tumorsphere-formation and high levels of β-catenin are accompanied with gain-of-function of stem cell-like properties, such as high levels of ALDH1 expression [35]. In all stages on the TMA, both ALDH1 and β-catenin were overexpressed compared to bulk cancer cells. The positive staining suggests that β-catenin may serve as a promising marker for prognosis of colon cancer.

**Figure 5:**
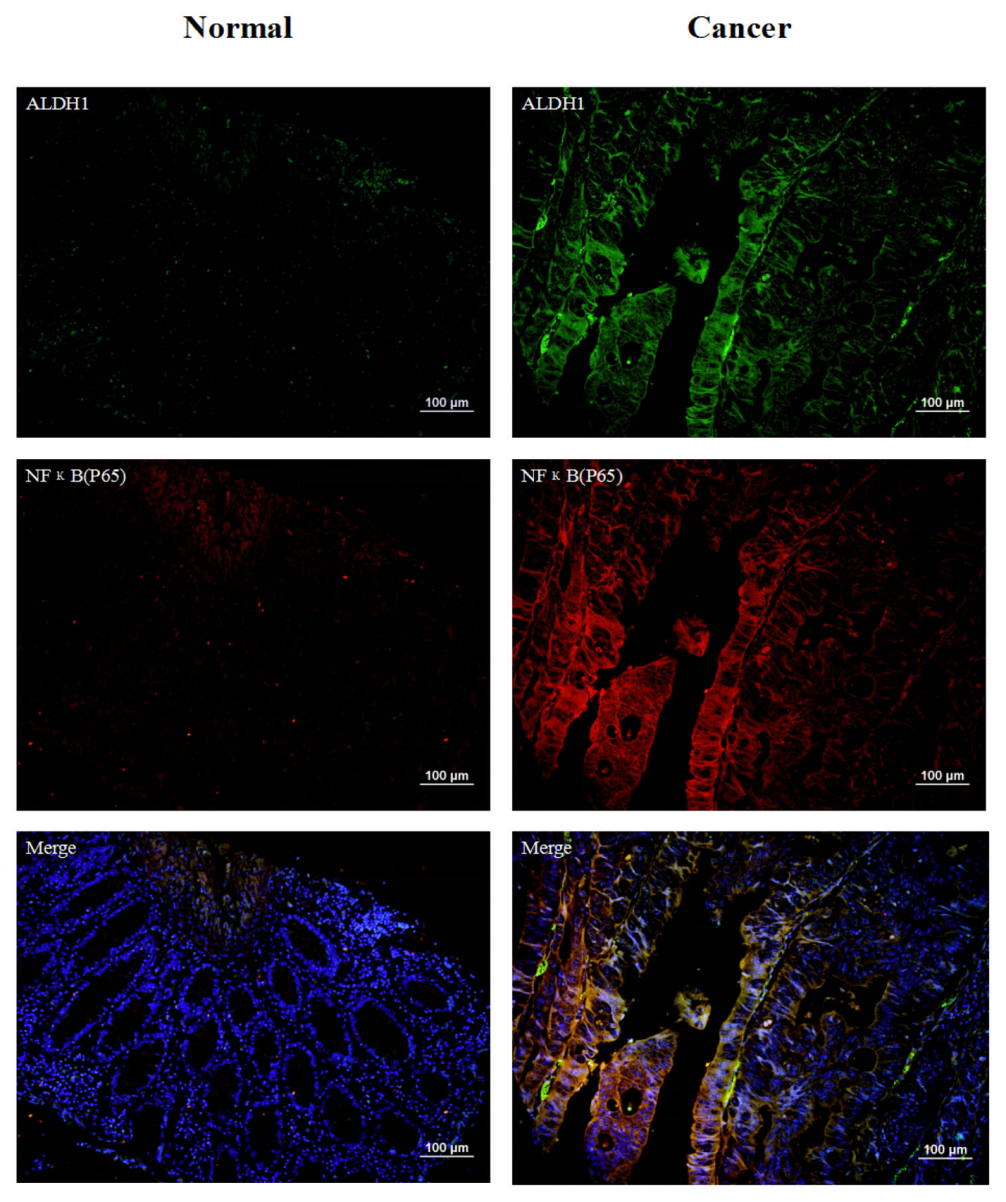
Immunofluorescence double staining with ALDH1 (green) and NFκB(p65) (red) in human normal colon tissue and colon cancer. DAPI represent the nucleus of the cells. There is no expression of ALDH1 and NFκB(p65) in normal tissues. NFκB(p65) is highly expressed in the cytoplasm. The overlaps have been shown in the merged images. Magnification 200x, scale bars=100 μm.

NFκB is known to be important in tumor promotion and progression [36,37]. Moreover, a previous study demonstrated that nuclear factor-κB (NFκB) activation is one of the factors resulting in chemotherapeutic agent’s resistance in tumors [38]. NFκB appeared in our network A which is involved in cell to cell signaling although not directly detected by mass spectrometry. By inducing transcription and crosstalk through signaling mediators, NFκB is an important element of tumorigenesis. Staining images are shown in Figure 5. In Table 2, there are 94 cases which have NFκB overexpression in 155 cases of colon cancer. The expression of NFκB (p65) highly overlaps with ALDH1 (93 cases) (Table 3). Among these 93 cases, we quantified the percentage of cell overlap to be around 81.4% (Table S6). The high expression and overlap indicate NFκB is a possible oncogene candidate related to colon cancer stem cells.

**Figure 6:**
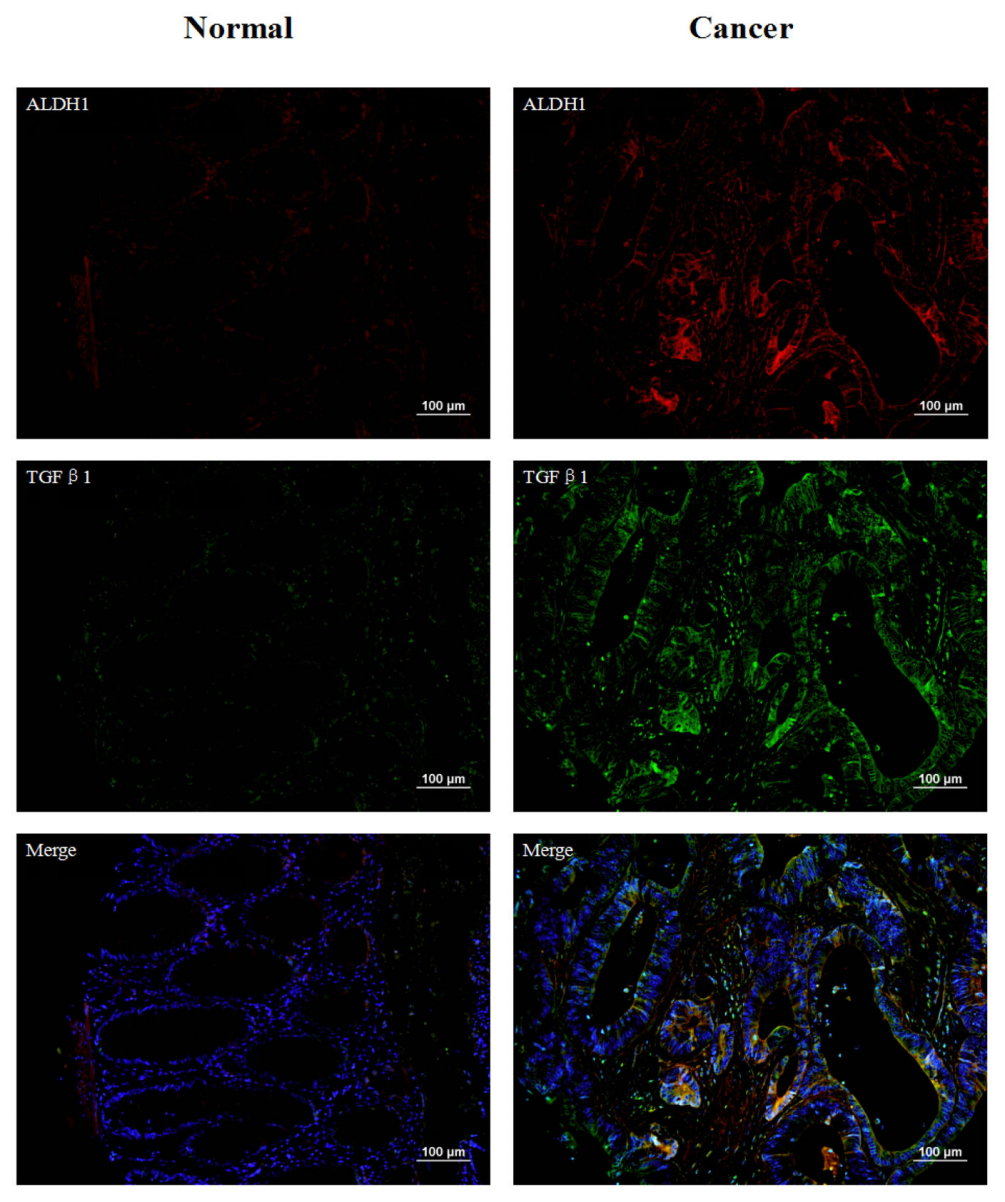
Immunofluorescence double staining with ALDH1 (red) and TGFβ1 (green) in human normal colon tissue and colon cancer. DAPI staining represents the nucleus of the cells. The expressions of ALDH1 and TGFβ1 are significantly increased in cancer tissues compared to normal tissues. TGFβ1 shows strong cell membranes and extracellular positive staining. Magnification 200x, scale bars=100 μm.

TGFβ1 is a natural pleiotropic growth factor that has the ability to regulate diverse biologic processes for a variety of cell types [39,40]. TGFβ1 has been reported as having great potential to induce invasion in several cancer types, including colon cancer cells. In our study, TGFβ1 is detected by mass spectrometry (Table S2). It is also shown in network B which is related to organismal injury and abnormalities. TGFβ1 staining images of normal tissues and cancer tissues are shown in Figure 6. The staining of TGFβ1 showed 65.8% strong cytoplasmic positivities in tumor cells (Table 2). There are 98 cases showing the overlap between ALDH1 and TGFβ1, while the overlap of ALDH1 and TGFβ1 positive cells among these 98 cases is around 76.3% (Table 3, S6). As in previous studies, during tumor progression, the existence of TGFβ1 in tumors is important to cancer invasion and metastasis through stimulating TGFβ1-responsive cell migration [41].

To eliminate leukocytes from ALDH+ cells, we performed double immunofluorescence staining of ALDH1 with CD45 (leukocyte common antigen) on a tissue microarray which contains 24 cases (20 cancer tissue cases and 4 normal tissue cases). The results show that CD45 has little expression in normal tissue and late-stage cancer tissue, although there is more expression in early-stage cancer tissue (Figures S1 and S2). We found that there was little or no co-expression with ALDH1+ cells so that the cells microdissected in this study were not leukocytes but colorectal cancer cells.

**Figure 7:**
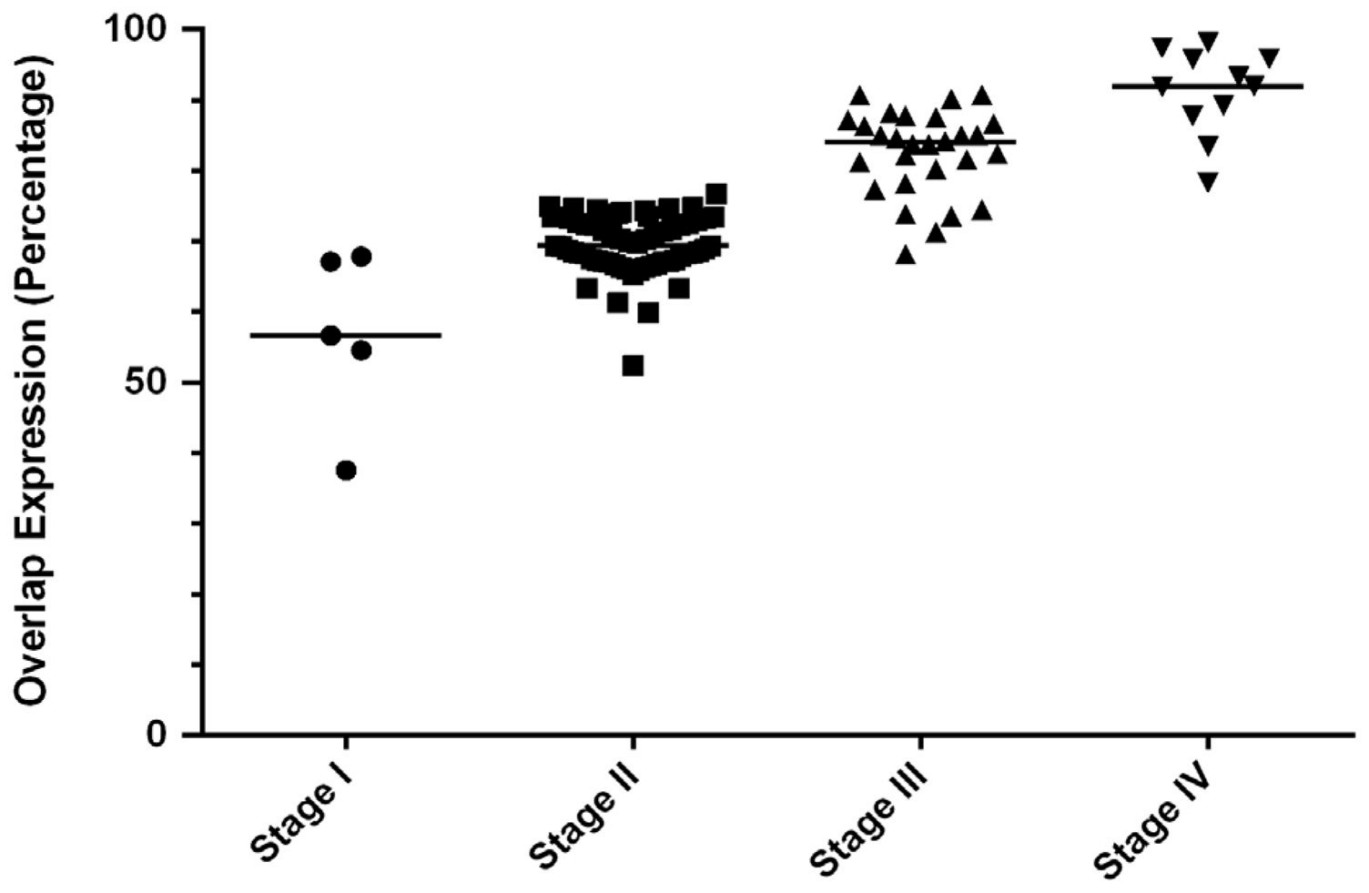
Association between ALDH1+ & β-catenin+ overlap expression and stages. X axis represents different stages. And Y axis represents the percentages of the overlaps. All overlap cases (n=92) and the medians are shown in this figure.

In order to observe the association between stages and expressions of these potential markers, we performed statistical analysis to find the median of protein expressions on different stages. With the advance of the stages, the medians of different protein expressions continuously increase. However, there is no significant difference between the expressions of different stages. We performed the same statistical analysis to study the correlation between stages and overlaps. Though the medians of overlaps between ALDH1 expressions and candidate markers expressions increase along with the developing stages, there is no statistically significant difference. The association between ALDH1 & β-catenin overlap and stages data is shown in Figure 7; plots for the other proteins are not shown.

In this study, we tested several markers other than β-catenin, NFkB (p65) and TGFβ1. We performed validation on the expression of CD24, CD44, CD90 and CD133 which are often found in cancer stem cells. No significant difference of expression of these markers was observed between cancer tissue and normal tissue (data not shown).

### Comparison with previous CRC-related studies

We compared the proteomic profile of ALDH1+ colorectal cancer stem cells and ALDH1− cells identified in this study with other CRC-related studies reported previously. Most of these CRC-related proteomics studies were performed using cell lines (i.e., CRC cell lines and normal colon cell line) [42–44] or bulk tissue (such as colon cancer tissue and adjacent normal tissue) [44–47] and thus the proteins expressed would be expected to be very different. For example, Di Palma et al. [43] applied FACS analysis to sort a limited number of colon stem cells extracted from mouse intestine which led to the identification of 1085 proteins. A recent proteomic analysis [46] of paired colorectal cancer and adjacent normal tissues from CRC patients identified 948 proteins in total, of which 184 proteins were differentially expressed (P<0.05, fold change >1.5) between tumor and non-tumor tissues. Among these proteins, cancer associated proteins such as FN1, TNC, DEFA1 and ITGB2 were found upregulated [46]. Using a proteomic analysis of minute amounts of colonic biopsies by enteroscopy sampling, Liu et al. identified 2620 proteins between cancer mucosa and adjacent normal colorectal mucosa, of which 195 proteins were differentially upregulated in cancer tissues [47].

Proteomic analysis based on specific cell subpopulations in CRC has been rarely reported. In this study, we used ALDH1 (a colorectal CSC marker) to separate specific subpopulations, i.e., ALDH1+ CSCs and ALDH1− cells, from three CRC patient specimens at stage III, where we were able to identify 1927 proteins across the three patient-matched specimens, of which 134 differentially expressed proteins (P<0.05, fold change >2) were significantly associated with ALDH1+ CSCs in colorectal cancer. Among these significant proteins, FTL, SDHA, HNPNPA, DHX family and MYH family were also reported to be associated with colon cancer stem cells in a proteomic study based on the colonosphere cultures derived from resection specimens of liver metastases in a patient with colon cancer [48]. We investigated a comprehensive comparison of proteome profiles between ALDH1+ CSC and ALDH1− subpopulations procured directly from local CRC tissues which could provide an improved means to uncover unique signatures associated with colorectal CSCs that are involved in regenerating the tumor as well as molecules involved in tumor initiation and progression of colorectal cancer.

## Conclusion

In this study, the ALDH1+ and ALDH1− cells were derived from FFPE tissues of three patients using immuno-LCM. Based on LC-MS/MS analysis, 1927 proteins were identified, of which 134 proteins were significantly differentially expressed in the three patients by at least a 2-fold change in ALDH1+ colon cancer cells compared to ALDH1− cells. Ingenuity Pathway analysis (IPA) was used to analyze 134 differentially expressed proteins relevant to organismal injury and abnormalities and cell-to-cell signaling pathways. The TMA immunofluorescence data of three selected candidate biomarkers, β-catenin, TGFβ1 and NFκB verified that they are highly overexpressed in colon cancer stem cells and negative in bulk cancer cells. These proteins were selected for TMA analysis based on the IPA pathway analysis and their known important roles in cancer progression. This method represents an alternative means of identifying important pathway proteins in cancer stem associated cells. The proteins studied herein may be possible candidates as targets in colon cancer therapy and for detection.

## Supplementary Material

1

## Figures and Tables

**Table 1:** Clinical pathologic characteristics of the patients with colon cancer (n=155) in the TMA set.

**Median age**	55 y (range,16–86 y)
**Gender (male/female)**	108 (69.7%)/47 (30.3%)
**Stage**	
I	6 (3.9%)
II	92 (59.4%)
III	43 (27.7%)
IV	14 (9.0%)
**Pathological grade**	
G1	47 (30.3%)
G2	47 (30.3%)
G3	40 (25.8%)
Not assessed	21 (13.6%)

**Table 2:** Summary of immunofluorescence data of β-Catenin, NFκB(p65), TGFβ1 and ALDH1 expression in normal tissues (n=15) and cancer tissues (n=155).

Tissue	No. of cases	ALDH1_pos	β-Catenin_pos	TGFβ1_pos	NFκB(p65)_pos
Normal	15	0	0	0	0
**Stage**					
I	6	6 **(100%)**	5 (83.3%)	4 (66.7%)	4 (66.7%)
II	92	72 **(78.3%)**	66 **(71.7%)**	58 (63.0%)	49 (53.3%)
III	43	38 **(88.4%)**	31 **(72.1%)**	29 (67.4%)	29 (67.4%)
IV	14	13 (**92.9%)**	11 **(78.6%)**	11 (78.6%)	12 (85.7%)
**Grade**					
G1	47	41 (**87.2%)**	35 (**74.5%)**	33 (70.2%)	28 (59.6%)
G2	47	43 (**91.5%)**	37 (**78.7%)**	30 (63.8%)	32 (68.1%)
G3	40	29 (**72.5%)**	26 (**65.0%)**	27 (67.5%)	23 (57.5%)
**Not assessed**	21	16 (**76.2%)**	15 (**71.4%)**	12 (57.1%)	11 (52.4%)
**Total of tumors**	155	129 (**83.2%)**	113 **(72.9%)**	102 **(65.8%)**	94 (60.6%)

**Table 3:** Percentage of cases with both expressions of ALDH1 and candidate markers.

	No. of cases	ALDH1 & β-Catenin	ALDH1 & NFκB(p65)	ALDH1 & TGFβ1
Normal	15	0	0	0
Stage I	6	5 (83.3%)	4(66.7%)	4 (66.7%)
Stage II	92	62 (67.4%)	48 (52.2%)	55 (59.8%)
Stage III	43	28 (65.1%)	29 (67.4%)	28 (65.1%)
Stage IV	14	11 (78.6%)	12 (85.7%)	11 (78.6%)
Total	155	106 (68.4%)	93 (60.0%)	98 (63.2%)
