# Supplementary material for: Protein Markers Associated with an ALDH Sub-Population in Colorectal Cancer
Source: J Proteomics Bioinform. Author manuscript; Available in PMC 2017 Oct 1. (PMC5423664; doi:10.4172/jpb.1000412)
Supplement: 1 [file NIHMS823341-supplement-1.pdf]

Figure S1

A)

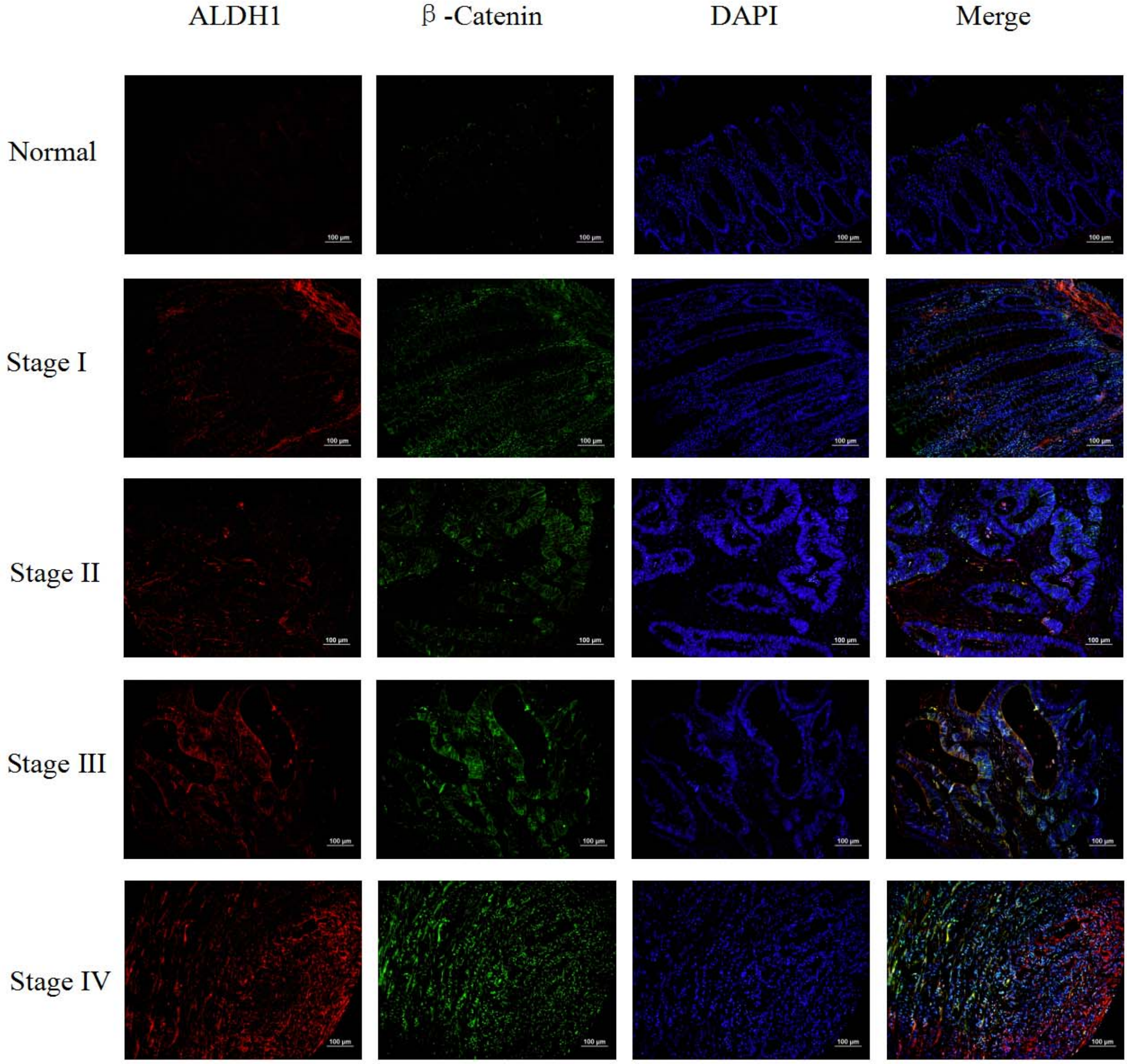

B)

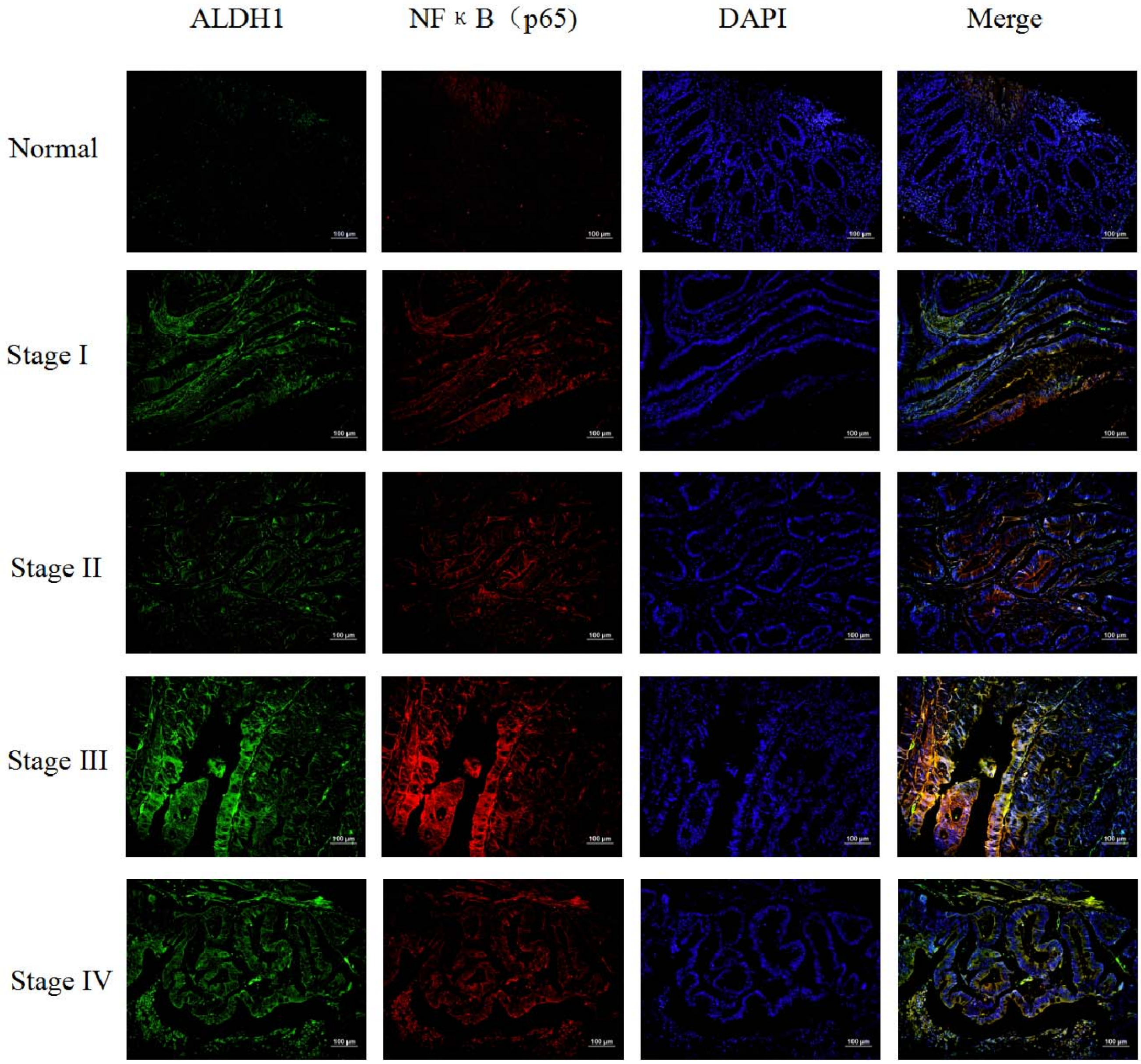

ALDH1
NFκB（p65)
DAPI
Merge
Normal
Stage I
Stage II
Stage III
Stage IV
100 μm

C)

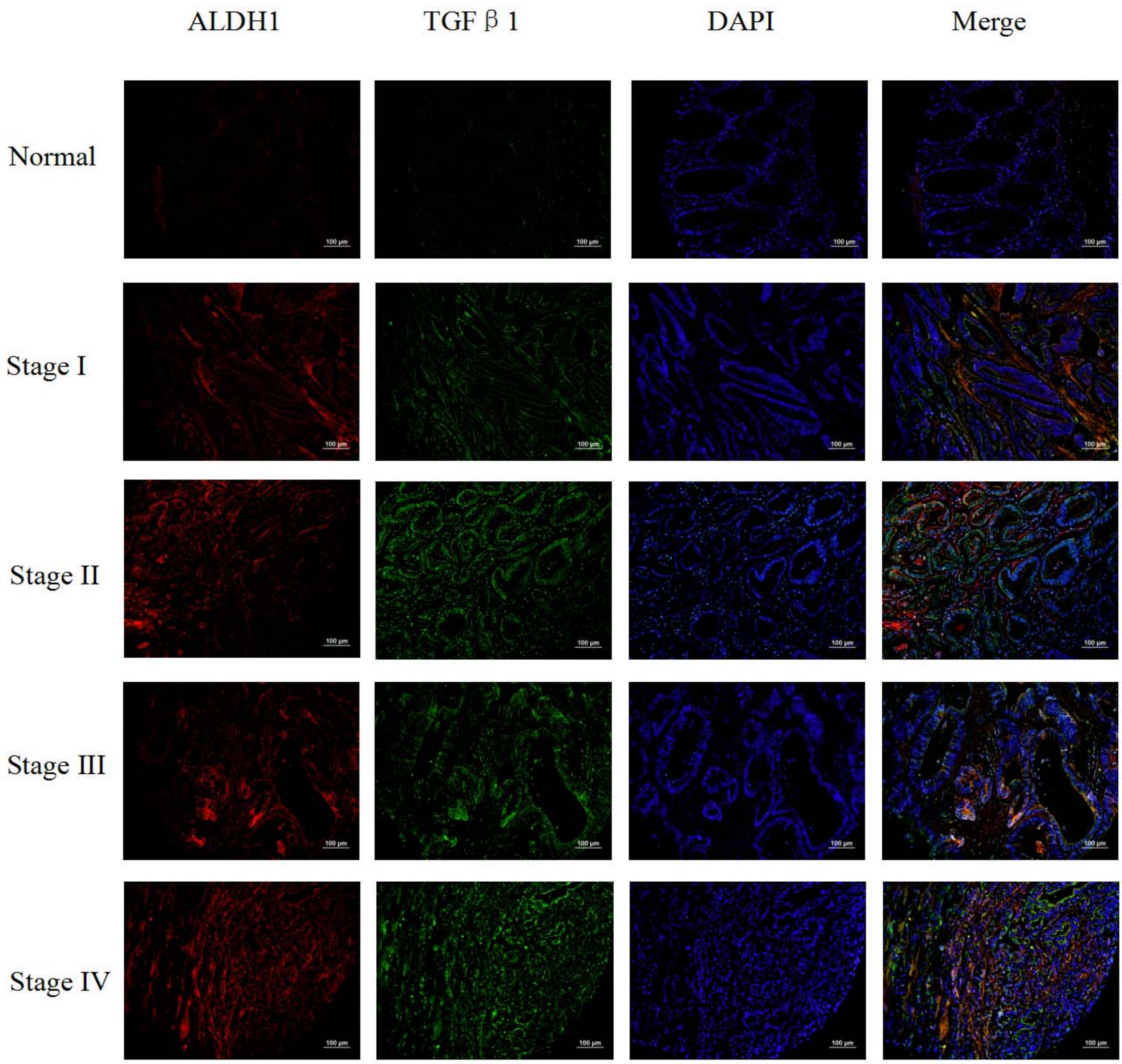

Figure S1. A) Immunofluorescence double staining with ALDH1 (red) and β-catenin (green) in human normal colon tissue and colon cancer micro-tissue arrays of stage I, II, III and IV. DAPI represent the nucleus of the cells. Overall, the expressions of ALDH1 and β-catenin are negative in normal tissue. ALDH1 is positive on cell membranes. However, both of them are overexpressed in cancer tissue. β-catenin shows strong nucleus positive and highly overlapped with ALDH1. Magnification 200×, scale bars = 100 μm. B) NFκB(p65) (red) is highly expressed in cytoplasm. C) TGFβ1 (green) shows strong cell membranes and extracellular positive.

Figure S2

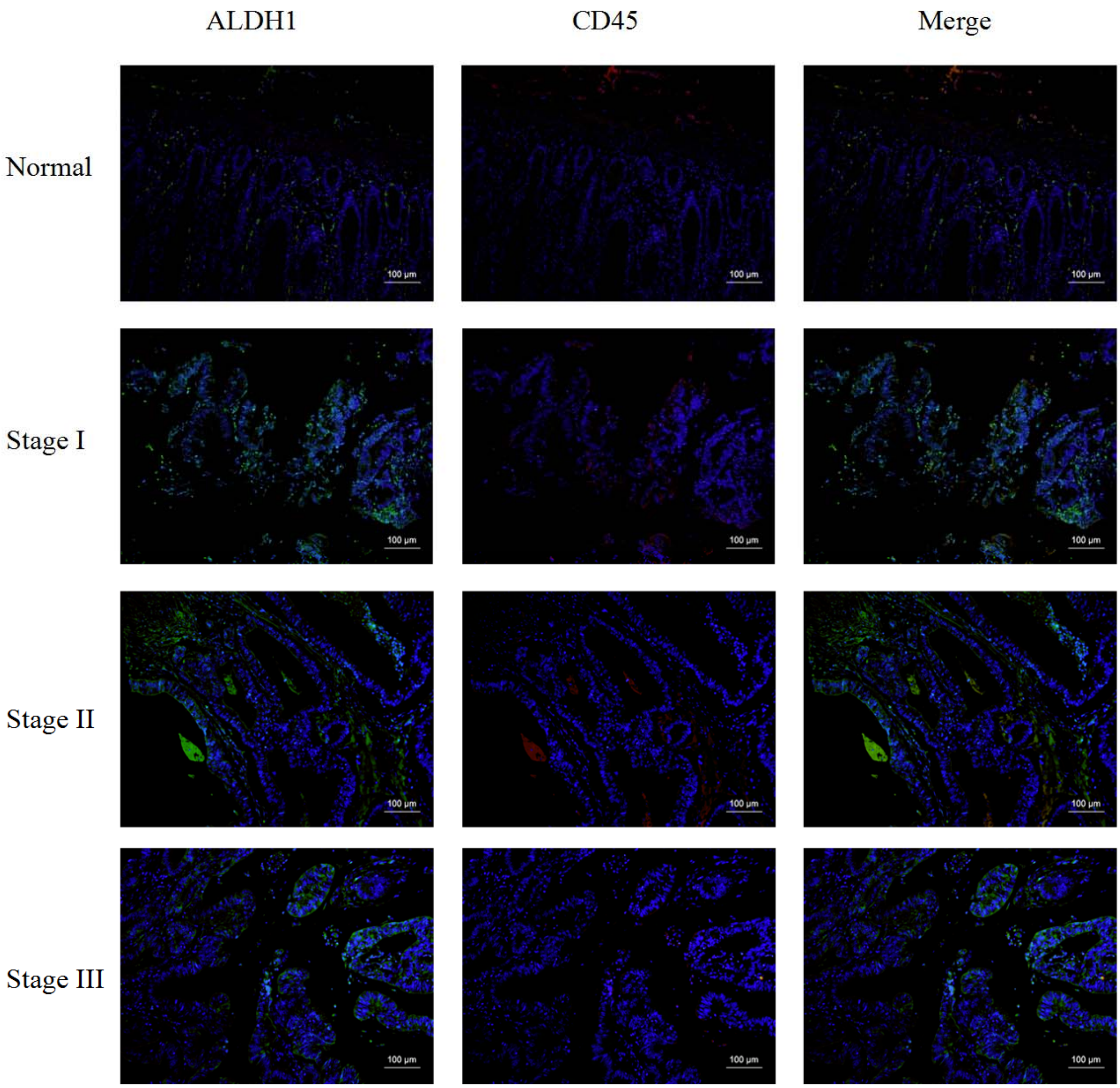

Figure S2. Immunofluorescence double staining with ALDH1 (green) and CD45 (red) in human normal colon tissue and colon cancer micro-tissue arrays of stage I, II and III. DAPI represent the nucleus of the cells. The expressions of ALDH1 and CD45 are negative in normal tissue. ALDH1 is positive on cell membranes in cancer tissue of all stages. However, CD45 has more expression in early-stage cancer tissue compared to late-stage cancer tissue. CD45 shows positive expression in connective tissue and has no overlap with ALDH1. Magnification 200×, scale bars = 100 μm.

Table S1. Patients' information. Characteristics of patient colon cancer tissues used in this study.

No.	Age	Sex	Stage	Sample ID	Pathological Diagnosis
1	75	Male	IIIA	ALDH1+_1	Adenocarcinoma
				ALDH1-_1	Adenocarcinoma
2	63	Male	IIIC	ALDH1+_2	Adenocarcinoma
				ALDH1-_2	Adenocarcinoma
3	68	Female	III	ALDH1+_3	Adenocarcinoma
				ALDH1-_3	Adenocarcinoma

Table S4. ALDH isoforms were detected by mass spectrometry. 13 isoforms are listed in this table. The fold change was calculated as the ratio of the normalized spectral abundance of each protein between ALDH1+ colon cancer cells and ALDH1- cells in the three patients respectively.

Accession	Protein Name	Gene	Ratio of ALDH1+ to ALDH1-		
			Sample pair		
			#1	#2	#3
C9J8Q5	Succinate-semialdehyde dehydrogenase, mitochondrial	ALDH5A1	NC[a]	ND[b]	ND
F8VS02	Alpha-aminoadipic semialdehyde dehydrogenase	ALDH7A1	NC	ND	ND
O94788	Reti0l dehydrogenase 2	ALDH1A2	ND	NC	ND
P00352	Reti0l dehydrogenase 1	ALDH1A1	ND	ND	NC
P05091	Aldehyde dehydrogenase, mitochondrial	ALDH2	ND	NC	0.33
P30038	Delta-1-pyrroline-5-carboxylate dehydrogenase, mitochondrial	ALDH4A1	ND	ND	0[c]
P30837	Aldehyde dehydrogenase X, mitochondrial	ALDH1B1	0.44	7.50	0
P49189	4-trimethylaminobutyraldehyde dehydrogenase	ALDH9A1	ND	NC	1.33
P49419	Alpha-aminoadipic semialdehyde dehydrogenase	ALDH7A1	ND	NC	ND
P49419-3	Isoform 3 of Alpha-aminoadipic semialdehyde dehydrogenase	ALDH7A1	NC	ND	ND
P54886	Delta-1-pyrroline-5-carboxylate synthase	ALDH18A1	ND	NC	0.37
P54886-2	Isoform Short of Delta-1-pyrroline-5-carboxylate synthase	ALDH18A1	NC	NC	ND
Q02252	Methylmalonate-semialdehyde dehydrogenase [acylating], mitochondrial	ALDH6A1	ND	NC	NC

Note: [a]NC, not calculable (number/0), which means that the protein was only identified in ALDH1+ cells. [b]ND, not detected. [c]0 represents that the protein was identified only in ALDH1- cells.

Table S5. Significantly expressed proteins identified between ALDH1+ colon cancer cells and ALDH1- cells, which were involved in the networks of organismal injury and abnormalities and cell to cell signaling. 40 proteins are involved in these two networks.

Accession	Protein Name	Gene	Ratio of ALDH1+ to ALDH1- Sample pair #1	#2	#3	p-value	Location
			Cell-to- Cell Signaling				
P24752	acetyl-CoA acetyltransferase 1	**ACAT1**	3.19	NC[a]	NC	1.56E-05	Cytoplasm
Q16186	adhesion regulating molecule 1	**ADRM1**	NC	NC	NC	5.89E-08	Plasma Membrane
P05023-3	ATPase, Na+/K+ transporting, alpha 1 polypeptide	**ATP1A1**	NC	NC	NC	4.42E-07	Plasma Membrane
P01024	complement component 3	**C3**	0[b]	0.27	0.10	1.74E-07	Extracellular Space
H3BVI7	cadherin 1	**CDH1**	NC	NC	NC	4.83E-04	Plasma Membrane
B4DGU4	catenin beta 1	**CTNNB1**	NC	NC	NC	1.04E-05	Nucleus
Q92841-1	DEAD-box helicase 17	**DDX17**	NC	NC	NC	5.04E-06	Nucleus
P17661	desmin	**DES**	0.32	0	0.13	7.26E-05	Cytoplasm
Q9UGM3-7	deleted in malignant brain tumors 1	**DMBT1**	NC	NC	NC	2.53E-05	Plasma Membrane
P06396-2	gelsolin	**GSN**	0.	0.20	0	6.57E-06	Extracellular Space
P10809	heat shock protein family D (Hsp60) member 1	**HSPD1**	2.15	3.34	NC	9.51E-03	Cytoplasm
Q8WWA0	intelectin 1 (galactofuranose binding)	**ITLN1**	NC	NC	NC	2.56E-03	Plasma Membrane
P14923	junction plakoglobin	**JUP**	28.35	NC	2.03	7.98E-04	Other
P17931	lectin, galactoside-binding, soluble, 3	**LGALS3**	NC	8.48	NC	4.61E-10	Extracellular Space
P26038	moesin	**MSN**	0.41	0.09	0.08	2.14E-04	Plasma Membrane
P35749-4	myosin, heavy chain 11, smooth muscle	**MYH11**	0.05	0	0	9.91E-06	Cytoplasm
O00151	PDZ and LIM domain 1	**PDLIM1**	NC	NC	NC	2.76E-07	Cytoplasm
P35232	prohibitin	**PHB**	2.94	NC	23.67	3.26E-07	Nucleus
P20618	proteasome subunit beta 1	**PSMB1**	NC	NC	NC	2.12E-05	Cytoplasm
P28072	proteasome subunit beta 6	**PSMB6**	NC	NC	NC	2.75E-03	Nucleus
P05387	ribosomal protein, large, P2	**RPLP2**	3.07	NC	NC	6.46E-05	Cytoplasm
P17987	t-complex 1	**TCP1**	NC	NC	NC	4.27E-04	Cytoplasm
P18206-2	vinculin	**VCL**	0.18	0.14	0	9.76E-07	Plasma Membrane
			organismal injury and abnormalities				
P62736	actin, alpha 2, smooth muscle, aorta	**ACTA2**	0	0.35	0	4.70E-05	Cytoplasm
P00966	argininosuccinate synthase 1	**ASS1**	NC	NC	NC	8.97E-03	Cytoplasm
P01024	complement component 3	**C3**	0	0.27	0.10	1.74E-07	Extracellular Space
P08572	collagen, type IV, alpha 2	**COL4A2**	0	0	0.18	2.58E-06	Extracellular Space
E9PP21	cysteine and glycine rich protein 1	**CSRP1**	0	0	0	6.08E-04	Nucleus
P15924	desmoplakin	**DSP**	NC	NC	61.96	2.18E-04	Plasma Membrane
F6RFD5	destrin (actin depolymerizing factor)	**DSTN**	0	0	0	4.49E-04	Cytoplasm
Q06210-2	glutamine--fructose-6-phosphate transaminase 1	**GFPT1**	NC	NC	NC	1.47E-05	Cytoplasm
P09601	Heme oxygenase 1	**HMOX1**	ND[c]	ND	NC	3.32E-01	Cytoplasm
P08648	Integrin alpha-5	**ITGA5**	0	ND	ND	7.03E-02	Plasma Membrane
P09874	Poly [ADP-ribose] polymerase 1	**PARP1**	ND	ND	0.50	7.69E-01	Nucleus
P11831	Serum response factor	**SRF**	ND	NC	ND	3.32E-01	Nucleus
H0YCU9	transgelin	**TAGLN**	NC	NC	NC	9.73E-05	Cytoplasm
O43294-2	Isoform 2 of Transforming growth factor beta-1-induced transcript 1 protein	**TGFB1I1**	NC	ND	ND	6.28E-02	Extracellular Space
B4DIT7	transglutaminase 2	**TGM2**	0	0.15	0	3.48E-06	Cytoplasm
Q07157	Tight junction protein ZO-1	**TJP1**	ND	ND	NC	3.32E-01	Plasma Membrane
B0YJC4	Vimentin	**VIM**	ND	NC	ND	6.31E-02	Cytoplasm

Note: [a]NC, not calculable (number/0), which means that the protein was only identified in ALDH1+ cells. [b]0 represents that the protein was identified only in ALDH1- cells. [c]ND, not detected.

Table S6. Positivity of cells with an overlap between ALDH1 and other potential markers. Among previous overlapped cases, cells with the ALDH1 positive and candidate makers positive staining have been counted to calculate the percentages.

	**ALDH1 & β-Catenin**	**ALDH1 & NFκB(p65)**	**ALDH1 & TGFβ1**
**Stage I**	37.6%-67.9%	52.4%-84.3%	41.7%-79.9%
**Stage II**	33.4%-76.7%	43.4%-79.7%	33.6%-87.7%
**Stage III**	52.4%-90.7%	32.7%-82.6%	37.9%-95.6%
**Stage IV**	43.3%-98.2%	55.4%-92.2%	55.1%-93.2%
**Overlap**	74.8%	81.4%	76.3%
